# Colovesical fistula in a young adult due to sigmoid colon diverticulitis undetected in computed tomography: Case report and review of literature

**DOI:** 10.1016/j.amsu.2021.102658

**Published:** 2021-08-05

**Authors:** Francisco Marcos da Silva Barroso, Carolina Augusta Dorgam Maués, Gustavo Lopes de Castro, Renato da Silva Galvão, José Paulo Guedes Saint Clair, Laura Riberio Aref Kzam

**Affiliations:** a Urology Service at Hospital e Pronto-Socorro 28 de Agosto, Avenida Mário Ypiranga, 1581, Adrianópolis, Manaus, Amazonas, 69057-000, Brazil; b General Surgery Service at Hospital e Pronto-Socorro 28 de Agosto, Avenida Mário Ypiranga, 1581, Adrianópolis, Manaus, Amazonas, 69057-000, Brazil; c Faculty of Medicine of the Federal University of Amazonas (UFAM), Rua Afonso Pena, 1053, Praça 14 de Janeiro, Manaus, Amazonas, 69020-160, Brazil; d General Surgery Service at Getúlio Vargas University Hospital (HUGV), Avenida Apurinã, 4 - Praça 14 de Janeiro, Manaus, Amazonas, 69020-170, Brazil

**Keywords:** Fistula, Colovesical fistula, Diverticulosis, Urinary bladder, Sigmoid colon, Enterectomy

## Abstract

**Introduction:**

Colovesical fistula is the pathological communication between the colon and urinary bladder. It is related with high morbimortality rate and it is and uncommon complication of diverticulosis in young adults.

**Case presentation:**

We report a case of a 38-year-old Brazilian man with fecaluria and pneumaturia for eight months, whose colovesical fistula was undetectable in CT scan and with surgical management.

**Discussion:**

Diverticulosis is the main inflammatory condition causing colovesical fistulas and the sigmoid colon is the most common part involved. It is more prevalent in patients over 60 years old and in western countries due to low fiber diet.

**Conclusion:**

Colovesical fistula diagnosis is difficult, requiring high suspicious and proper investigation through good anamnesis, CT scan and also colonoscopy and cystoscopy when necessary.

## Highlights

• Colovesical fistula is an uncommon disorder in young adults and it is more frequently in western countries.
• Diverticulits is the main inflammatory condition causing enterovesical fistulas and the sigmoid colon the most common.
• Colovesical fistula diagnosis is difficult, requiring proper investigation through CT scan or other exams.
• Most cases of colovesical fistula are diagnosed during surgery approach and surgically managed.

## 1 Introduction

Colovesical fistula is the most common enterovesical fistula type, which is the existence of a pathological communication between the colon and urinary bladder [[1], [2], [3]]. Diverticular disease induces these conditions in 65–75% of the enterovesical fistula cases [1,2]. Enterovesical fistulas are associated with high morbimortality [3,4].

Diverticular disease affects about 10% of the western population due to poor fiber diet and it is more common in the sigmoid colon, representing 50–70% of fistula sites [3,5,6]. It is an infrequent complication of acute diverticulitis and affects individuals over 60 years [7]. Besides the inflammatory etiology, pelvic neoplasms are also related with this condition [3].

We report a case of colovesical fistula undetected in Computed Tomography (CT) induced by sigmoid diverticular disease in a 38-year-old male patient and with surgical management.

This case report is being reported in line with the SCARE 2020criteria [8].

## 2 Case presentation

A 38-year-old Brazilian man was admitted in our service with chief complaint of fecaluria and pneumaturia for eight months that started after an intense abdominal pain at the time. His occupation was teaching and he walked into the urology emergency by his own means. The patient evolved with repeated episodes of urinary infection associated with dysuria and hematuria. He was treated with several antibiotics with no resolution of the main complaint of fecaluria and pneumaturia.

On admission, the patient was stable, afebrile, acyanotic, anicteric, with normal vital signs and physical examination with globose, flaccid abdomen, painless, with no peritoneal irritation. After two days, he reported dysuria and mild pain in hypogastric area. He was treated with ceftriaxone and metronidazole with relief of dysuria and hypogastric pain, but persistence of fecaluria and pneumaturia. He reported no surgical history, drug allergy or similar disorder in his family, including relevant genetic information and psychosocial history.

**Fig. 1:**
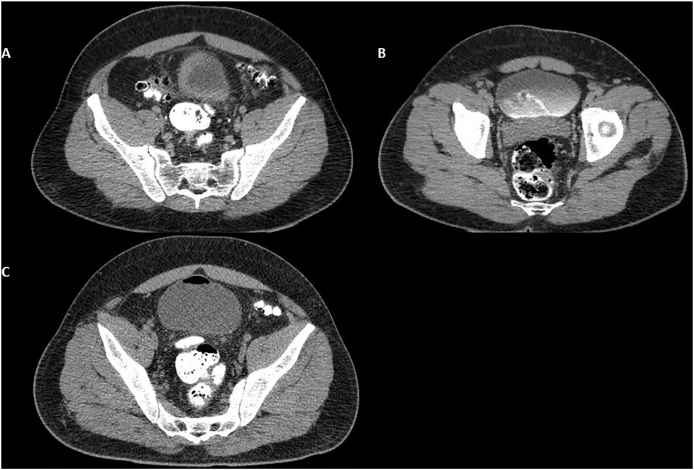
**A.** CT scan with oral contrast showing sigmoid colon contrasted but no evidence of contrast overflow to the bladder. **B.** CT scan after intravenous contrast with no evidence of communication between the urinary bladder and sigmoid colon, also it is possible to visualize the contrast flush from left ureter to the urinary bladder. **C.** New CT scan with oral contrast with no evidence of contrast overflow from the sigmoid colon to the urinary bladder.

Laboratory tests revealed normal serum values, including renal function (WBC 11.360; Hemoglobin 12.4; Urea: 40; Creatinine: 1.3). The patient was submitted to an abdominal and pelvic CT scan with oral and intravenous contrast that showed no evidence of enterovesical fistula. After six days, a new CT scan with oral contrast was performed but no evidence of fistula was detected in the exam (Fig. 1).

**Fig. 2:**
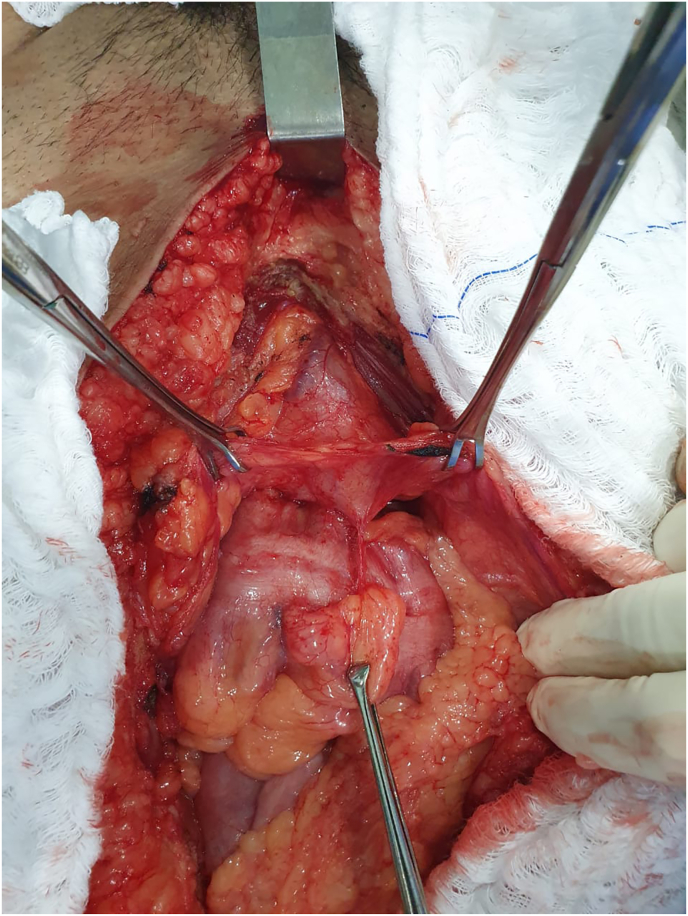
Adherences between the sigmoid colon and the urinary bladder found during surgical approach.

**Fig. 3:**
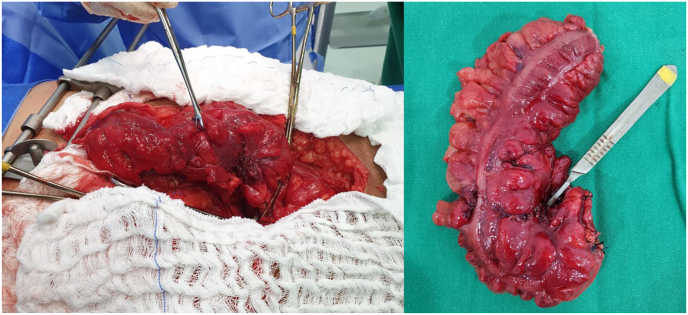
**A.** Necrotic tissue in the sigmoid colon after dissection from the bladder. **B.** Enterectomy performed in the sigmoid colon due to several diverticula.

He underwent exploratory laparotomy with supra and infraumbilical median incision with a urologist and a gastrointestinal tract surgeon. During the procedure, firm adherences between sigmoid colon and urinary bladder was identified (Fig. 2). A careful dissection was performed separating the sigmoid colon from posterior wall of the bladder, which was with necrotic tissue later dissected and the bladder wall repaired in two layers with vicryl 4–0 and 2–0, respectively. An extensive necrosis area was present in sigmoid colon, associated with several diverticula. A rectosigmoidectomy of approximately 25 cm was performed with colostomy of the proximal segment (Fig. 3). The surgery was performed by a general surgeon with digestive tract surgery specialization and by a urologist.

The dissected tissue of posterior bladder wall and sigmoid segment were submitted to histopathological analysis that revealed unspecific chronic inflammatory process compatible with colovesical fistula and colovesical fistula with diverticulitis, respectively. No malignant lesion was identified.

In post-operative recovery, the patient evolved painless, with a Foley catheter with discreet pieces of a white-yellow colored mass from the bladder. The intervention was nicely tolerable by the patient and he was discharged 12 days after surgery and oriented to remove the catheter in twenty-first day post-surgery. He was referred to a specialized service for gastrointestinal tract reconstruction, which was performed about five months later. In once a month follow-up, he evolved with absence of pneumaturia and fecaluria.

## 3 Discussion

The presence of an abnormal communication between two different cavities is called fistula and when it involves the colon tract and the urinary bladder is defined as a colovesical fistula [1,2]. This type of fistula is usually related to inflammatory processes or neoplasm conditions. Diverticulosis is the main inflammatory condition causing colovesical fistulas and is considered relatively rare [5,6].

Other etiologies related to this condition are Chron's disease, infection, trauma, and radiation [2,3,6]. The usual mechanism between diverticulosis and colovesical fistula is its rupture due to diverticulitis or adherences and erosion directly through the contact of these structures [9,10].

Colovesical fistulas are frequently more common in elder people and western countries, fact explained by the poor fiber diet in these regions. In patients with less than 40 years, the incidence of diverticulosis is lower than 5%, at the same time people older than 80 years is 65%. Colovesical fistulas are more common between 65 and 75 years old, differing from our patient who was 38 years old [2,[5], [6], [7]].

Enterovesical fistula is more common between sigmoid colon and urinary bladder, almost 90–95% of the cases. There is a small predominance in men, probably due to anatomic structures positions as uterus [3].

The chief complaint of patients with colovesical fistulas are fecularia and pneumaturia. These symptoms are extremely related to the diagnosis of this condition and 71.4% of patients with pneumaturia and 51 % with fecaluria are diagnosed with colovesical fistula [6,9]. Secondary symptoms may involve dysuria, polaciuria, suprapubic pain and hematuria [2,3]. For this reason, colovesical fistulas are initially treated as urinary infection before its proper diagnostic. Our patient himself underwent the use of several antibiotics in his medical history.

The diagnosis of this condition requires high suspicious and proper investigation. Although it is difficult, CT scan, colonoscopy or cystoscopy are helpful to identify the fistula. Computed tomography has a high sensitivity to for the diagnosis of colovesical fistulas, with an accuracy of almost 90% [[10], [11], [12], [13], [14]].

In our case, the patient was first submitted to a CT scan with oral contrast but no evidence of fistula. He was then submitted to an intravenous and oral contrast CT, still without any sign of contrast overflow between sigmoid colon and urinary bladder. After six days, a new CT with oral contrast was performed showing the same results. Despite the CT sensitivity for the diagnosis of colovesical fistula, this condition was undetectable in our patient through this exam and only found during surgery approach.

The treatment of colovesical fistula is divided in two options: conservative and surgical management. Open and laparoscopic surgery have been used to resolve this pathological communication. It is aimed to resect the adherences between the colon and bladder with anastomose of the gastrointestinal tract and bladder reconstruction [13,15]. Most important complication of colovesical fistula is the occurrence of sepsis, which may lead to death in untreated patients with this condition.

## 4 Conclusion

Colovesical fistula is rare in young adults and it is mainly related to diverticulosis. This condition may presente with a variance of symptoms, especially pneumaturia and fecaluria. Although CT presents high accuracy in its diagnosis, it may not detect small fistulas. Most of the cases are surgically treated through intestinal reanastomose and bladder reconstruction. High suspicious and proper investigation are required in the existence of pneumaturia and fecaluria to diagnose colovesical fistula.

### 4.1 Patient perspective

The patient reported in the follow-up he was grateful for the problem solution after surgery and that he could go back to his teaching activities without any of previous difficulties.

## Declaration of competing interests

All authors declare no conflict of interests.

## Ethical approval

This study was exempt from ethnical approval.

## Source of funding

This paper did not receive any specific grant from funding agencies in the public, commercial, or not-for-profit sectors.

## Author contribution

Francisco Marcos da Silva Barroso: study concept, data collection, writing the paper. Carolina Augusta Dorgam Maués: study concept and data collection. José Paulo Guedes Saint Clair: study concept and data collection. Renato da Silva Galvão: study concept and data analysis. Gustavo Lopes de Castro: study concept, data collection, writing the paper and design. Laura Ribeiro Aref Kzam: writing the paper and design.

## Registration of research studies

1. Name of the registry:
2. Unique Identifying number or registration ID:
3. Hyperlink to your specific registration (must be publicly accessible and will be checked):

## Consent

Written informed consent was obtained from the patient for publication of this case report and accompanying images.

## Provenance and peer review

Not commissioned, externally peer reviewed.

## Declaration of competing interest

Each named author has no conflict of interest, financial or otherwise.
